# Dimethyl Fumarate Ameliorates Lewis Rat Experimental Autoimmune Neuritis and Mediates Axonal Protection

**DOI:** 10.1371/journal.pone.0143416

**Published:** 2015-11-30

**Authors:** Kalliopi Pitarokoili, Björn Ambrosius, Daniela Meyer, Lisa Schrewe, Ralf Gold

**Affiliations:** Department of Neurology, St. Josef Hospital, Ruhr- University of Bochum, Bochum, Germany; LMU Munich, GERMANY

## Abstract

**Background:**

Dimethyl fumarate is an immunomodulatory and neuroprotective drug, approved recently for the treatment of relapsing-remitting multiple sclerosis. In view of the limited therapeutic options for human acute and chronic polyneuritis, we used the animal model of experimental autoimmune neuritis in the Lewis rat to study the effects of dimethyl fumarate on autoimmune inflammation and neuroprotection in the peripheral nervous system.

**Methods and Findings:**

Experimental autoimmune neuritis was induced by immunization with the neuritogenic peptide (amino acids 53–78) of P2 myelin protein. Preventive treatment with dimethyl fumarate given at 45 mg/kg twice daily by oral gavage significantly ameliorated clinical neuritis by reducing demyelination and axonal degeneration in the nerve conduction studies. Histology revealed a significantly lower degree of inflammatory infiltrates in the sciatic nerves. In addition, we detected a reduction of early signs of axonal degeneration through a reduction of amyloid precursor protein expressed in axons of the peripheral nerves. This reduction correlated with an increase of nuclear factor (erythroid derived 2)-related factor 2 positive axons, supporting the neuroprotective potential of dimethyl fumarate. Furthermore, nuclear factor (erythroid derived 2)-related factor 2 expression in Schwann cells was only rarely detected and there was no increase of Schwann cells death during EAN.

**Conclusions:**

We conclude that immunmodulatory and neuroprotective dimethyl fumarate may represent an innovative therapeutic option in human autoimmune neuropathies.

## Introduction

Guillain-Barré syndrome (GBS) and chronic inflammatory demyelinating polyneuropathy (CIDP) represent a spectrum of heterogeneous disabling neuropathies pathogenetically characterized by an autoimmune reaction against specific components of the peripheral myelin sheath [1,2,3]. For both of them, a response rate of up to 50% to first-line immunmodulatory treatment has been reported whereas immunosuppressive agents are beneficial in two- thirds of CIDP patients at the expense of serious adverse effects [4,5]. Neuroprotective agents are not yet available, although disability seems to be mainly determined by the degree of axonal degeneration. This may occur very early in the disease progression, and may have no direct correlation to the degree of demyelination [6,7].

The animal model of experimental autoimmune neuritis (EAN) enables the study of electrophysiological characteristics, histological appearance and immunological features of acute peripheral autoimmune neuropathy. It can be induced in Lewis rats by the inoculation of susceptible strains with various peripheral nervous system (PNS) antigens, like myelin protein P2 emulsified in complete Freund's adjuvant (CFA) [8,9]. After immunization, autoantigen-specific lymphocytes in secondary lymphoid organs migrate to the PNS and in turn recruit and activate mostly macrophages, which represent the major cell population in the inflamed PNS [10]. Subsequent demyelination and axonal damage occur through direct phagocytic attack, T-cell- mediated cytotoxicity, damage from Th1 cytokines, free oxygen radicals, complement-dependent attack, and antibody-mediated functional impairment [11].

The second-generation oral dimethyl fumarate (DMF) is approved since 2013 for multiple sclerosis, on the grounds of strong immunmodulatory and putative neuroprotective effects with a favourable safety profile in two large phase III studies (DEFINE and CONFIRM) and the extension study ENDORSE [12,13].

Studies in experimental autoimmune neuritis (EAE), the mouse model for multiple sclerosis, showed that DMF and its primary metabolite, monomethyl fumarate (MMF) ameliorated EAE course and preserved myelin and axonal intergrity [14,15]. As putative neuroprotective mechanism the activation of the transcription factor Nrf-2 (nuclear factor (erythroid derived 2)-related factor 2) was observed. Nrf-2 in turn raises the levels of the antioxidant glutathione and downregulates the expression of inflammatory cytokines, chemokines, and adhesion molecules [15,16,17,18,19]. Fumarates can induce T cell apoptosis in vitro, resulting to a decrease of T-cells in peripheral blood in nearly all treated patients with psoriasis [20,21].

Autoimmune neuropathies seem to have many pathogenetic similarities to multiple sclerosis on the basis of T cell- and macrophages-mediated demyelination and axonal damage and the regulatory role of myelinating Schwann cells in analogy to oligodendrocytes in CNS [22]. Therefore, we investigated the effect of orally administered DMF in EAN as a novel neuroprotective treatment option for autoimmune peripheral neuropathies.

## Materials and Methods

### Antigens

The neuritogenic P2 peptide, corresponding the amino acids 53–78 of bovine myelin P2 protein, was synthesized by Dr. Rudolf Volkmer from Charité University (Berlin, Germany).

### Induction of EAN and assessment of clinical score

A total of 62 female Lewis rats, 6–8 weeks old, purchased from Charles River Co. (Sulzfeld, Germany) and weighing 160–180 g were used in the present study. All animals were anaesthetized by exposure to 1.5%–2.0% halothane in oxygen) and immunized by subcutaneous injection into the root of the tail of 250 μg P2_53-78_ peptide in PBS, emulsified in an equal volume of CFA containing 1 mg/ml *Mycobacterium tuberculosis* H37RA (Difco, Detroit, MI). Animals were weighed and scored for disease severity daily by two investigators. Disease severity was assessed clinically employing a scale ranging from zero to 10 originally described by Enders *et al*. [23]: 0 normal; 1 less lively; 2 impaired righting/limb tail; 3 absent righting; 4 atactic gait, abnormal position; 5 mild paraparesis; 6 moderate paraparesis; 7 severe paraplegia; 8 tetraparesis; 9 moribund; 10 death. All experiments were reviewed and approved by the North-Rhine-Westphalia authorities for animal experimentation (TVA 84–02.04.2014-A451).

### *In vivo* treatment with dimethyl fumarate

Dimethyl fumarate (Biogen Idec, Cambridge, USA) was dissolved in 0.08% methylcellulose in tap water and a total volume of 300 μl was administered twice daily by oral gavage starting from the day of immunization to day 23 p.i; control groups received similar volume of methylcellulose 0.08% by oral gavage twice daily. Rats were kept under standardized, pathogen free conditions at the local animal facility, Medical Faculty, Ruhr-University, Bochum, Germany. Food and water were given ad libitum to all animals.

The animals were randomly divided into the following groups: control group treated with methylcellulose 0.08% in tap water (n = 8), a 15 mg/kg body weight DMF-treated group (n = 8), a 30 mg/kg body weight DMF-treated group (n = 8) and a 45 mg/kg body weight DMF-treated group (n = 8).

### Nerve conduction studies

Nerve conduction tests were performed on the day before immunisation (-1) and on days 16 (maximum of natural disease course) and 23 (recovery) post-immunization. At each time point 10 rats pro group were tested for each group. The rats were anesthetized intraperitoneally (i.p.) with xylazine and ketamine (10mg/kg and 50mg/kg respectively). By examining amplitude and latencies of the evoked compound muscle action potentials (CMAPs) recorded from the feet, we assessed sciatic nerve motor conduction. Using a fully digital recording Keypoint apparatus (Dantec, Skovlunde, Denmark) and paired needle electrodes inserted at the sciatic notch (hip; proximal) or the popliteal fossa (distal), the sciatic nerve was stimulated with supramaximal rectangular pulses of 0.05-ms duration and the resulting CMAP was recorded from needle electrodes placed subcutaneously over the dorsal foot muscles. A ground electrode was placed between the distal stimulating electrode and the active recording electrode. To calculate the motor nerve conduction velocity (MNCV), the distance between stimulating cathodes was divided by the latency difference. Similarly, the persistence and minimum latency of 10 F-waves evoked by stimulation at the popliteal fossa were recorded for the right side [24,25,26]. Temperature differences were minimized by conducting the study as soon as the anaesthesia had taken effect and by warming the leg with a heating lamp.

### Histopathological assessment and immunohistochemistry

After transcardial perfusion with phosphate buffered saline (PBS, Gibco) on disease maximum, Day 16 post immunization (p.i.) the two sciatic nerves were dissected, the segments were embedded in Tissue-Tek OCT Compound and snap-frozen in liquid nitrogen. For histopathology assessment, tissue of six (n = 6) rats per group (methylcellulose-treated, 15 mg/kg and 45 mg/kg DMF) was sectioned (10 μm) on a cryostat (Leica Biosystems, Nussloch, Germany) and mounted on slides.

For the immunohistochemical staining, cryostat sections after fixation in acetone at 20°C for 10 minutes, were exposed to the mouse monoclonal antibodies (mAb) anti-rat 15-6A1 (Pan T-Cells CD3, 1:100, Hycultec), anti-rat ED1 (anti-CD68, macrophages, 1:100, Hycultec) and anti-APP (amyloid precursor protein) (MAB348SP, 1:100, Millipore) using the avidin-biotin technique (Dako ARK KIT for mouse primary antibody). Schwann cell detection was performed using the anti-S-100 antibody (Dako, 1:100) whereas for Schwann cell apoptosis we used the In Situ Cell Death detection Kit (POD, Roche). Axons were stained with NF200 (neurofilament 200) antibody (MAB1523, Abnova, 1:100). Specificity of the staining was also controlled on sections of peripheral lymphoid organs for T cells and macrophages and rat brain for amyloid precursor protein. The numbers of positive cells were counted at ×40 magnification for 12 sections per animal. The average results are expressed as cells per mm^2^ tissue section.

For identification of Nrf2 expression a staining using the antibody anti-Nrf2 (C-20, sc-722, 1:100, Santa Cruz Biotechnologies) was performed according to manufacturer’s protocol and DAPI (4',6' diamino-2-phenylindole·2HCl) was used for fluorescent staining of DNA. Fluorescent signals were detected using an inverted fluorescence microscope (BX51; Olympus, Tokyo, Japan) equipped with an Olympus DP50 digital camera. For assessment of Nrf2 staining, images (20x-magnification) of twelve transverse sections of the sciatic nerve from each animal were digitally generated (Cell imaging software). The percentage of the area with Nrf2 staining per section was determined using image analysis software (ImageJ). Omission of the primary antibodies served as negative control.

### Isolation of mononuclear cells from lymph nodes and spleen and FACS analyses

The inguinal lymph nodes and spleen were removed after transcardial perfusion with phosphate buffered saline (PBS, Gibco) on disease maximum (day 16 p.i.) under aseptic conditions. Single cell suspensions of mononuclear cells from individual rats were prepared separately (n = 6/group). We evaluated the frequency of CD4^+^ T cells, CD11b^+^ cells, CD4^+^CD11b^+^ dendritic cells (DCs), CD4^+^ CD25^+^ FoxP3^+^ regulatory T-cells (Tregs) and CD4^+^CD11b^-^MHCII^+^ plasmatocytoid DCs and the expression of IL-10 and IL-17 from MNC by fluorescence-activated cell sorting (FACS) staining. FACS analyses were performed with a FACS Canto II (BD Pharmingen, Heidelberg, Germany) machine and FlowJo software (Tree Star). Monoclonal antibodies purchased from BD Pharmingen or eBioscience were used to detect CD4, CD11b, CD25 and MHC-II in accordance with the manufacturers’ instructions. Intracellular staining for Foxp3 was performed using the Foxp3 Staining Set (eBioscience, San Diego, CA) and intracellular staining for IL-10 and IL-17 was performed according to the manufacturer’s instructions (BD Pharmigen and eBioscience).

### Statistical methods

All analyses were performed completely blinded with respect to treatment. Statistical analysis was performed by one-way analysis of variance (ANOVA) or Kruskal–Wallis test (Graph Pad Prism6, San Diego, CA, USA). Data are provided as mean ± SEM. Differences between pairs of groups were tested by Student’s *t*-test. As post-hoc tests Bonferroni's Multiple Comparison Tests were performed. A probability level (p-value) of *p < 0.05, **p < 0.005 and ***p < 0.0001 was considered to be statistically significant for all tests. All error bars represent SEM.

## Results

### Dimethyl fumarate ameliorates rat experimental autoimmune neuritis

**Fig 1:**
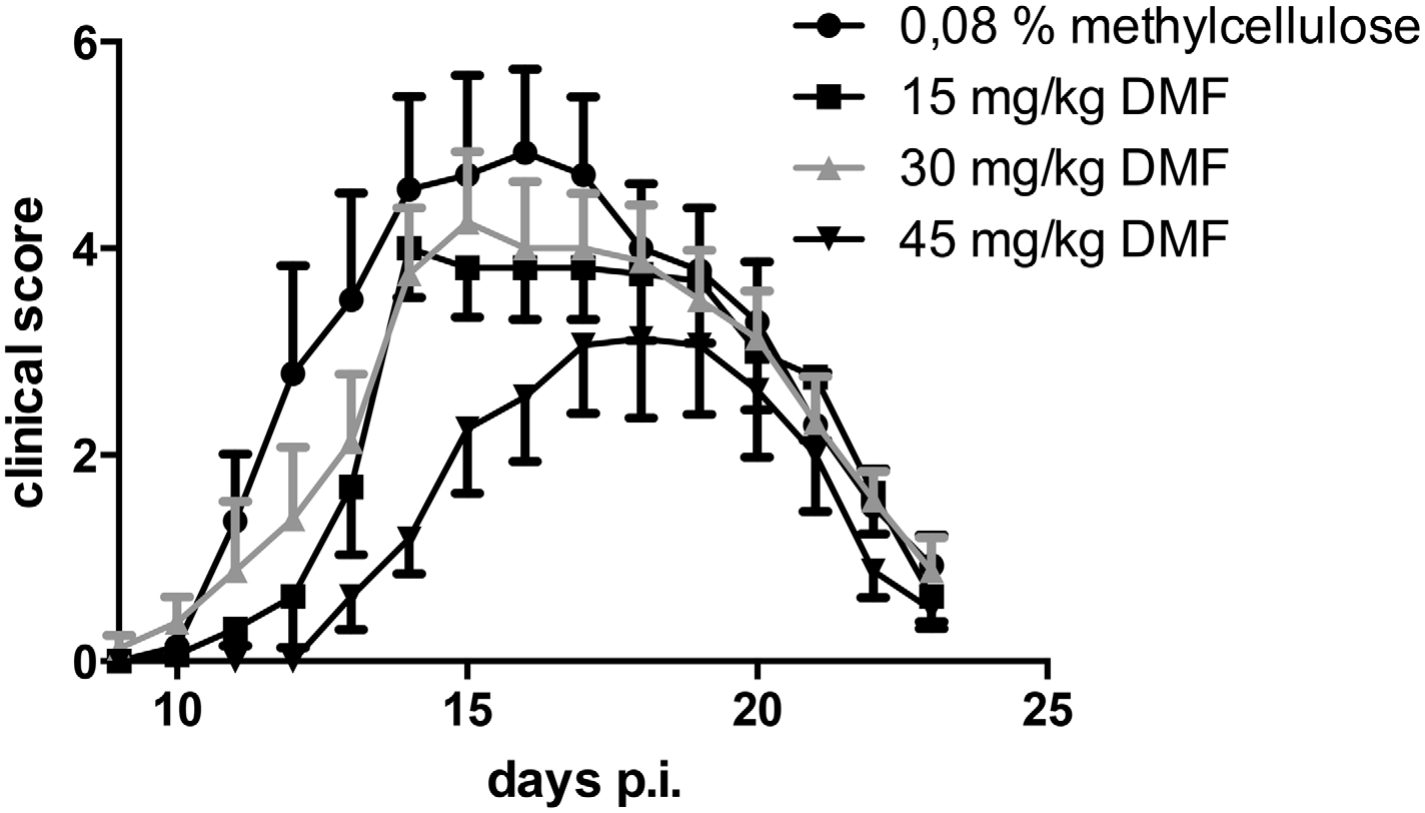
Clinical EAN course under dimethyl fumarate treatment. EAN was induced in Lewis rats by immunisation on day 0 with P2 peptide 53–78 plus CFA. Rats received DMF diluted in 0,08% methylcellulose in tap water at doses of 15 mg/kg, 30mg/kg and 45mg/kg twice daily from day 0 to day 23-post immunisation by oral gavage. Control rats received 0,08% methylcellulose in tap water only. Mean values and SEM are depicted, ROC Area under curve (AUC) 45mg/kg vs. methylcellulose, n = 8 * p<0,05. The experiment was repeated 2 times with similar results.

After immunization with P2 protein peptide 53–78, clinical signs of EAN started around day 11 p.i.. The incidence of EAN for the control group was 100% and the groups receiving DMF showed an incidence of 100% (15 mg/kg), 100% (30 mg/kg) or 87.5% (45 mg/kg) respectively (n = 8). Treatment with 15 and 30 mg/kg had no statistically significant effect on the clinical course of EAN. Treatment with 45 mg/kg DMF delayed the onset of clinical EAN by 2–3 days and reduced significantly the clinical signs of EAN when compared with methylcellulose-treated control rats (Fig 1, ROC AUC, area under curve, *p<0,05). A further experiment was performed with a group treated with 100mg/kg DMF (n = 8). This high dosage showed no clinical effects and no overt signs of toxicity (data not shown). There was a non-significant reduction of body weight of sham-treated controls during the course of active EAN as compared to rats treated with 45 mg/kg DMF (data not shown), in accord with our experience with this disease model [11].

### Dimethyl fumarate improves proximal and distal nerve conduction

As described in material and methods we performed electrophysiological measurements of the sciatic nerve at different stages of the clinical course in order to elucidate the mechanisms of EAN attenuation after DMF treatment. The following measures were evaluated: 1) axonal damage, as indicated by a synchronous reduction of CMAP amplitude after proximal and distal stimulation 2) degree of demyelination, as implied by conduction block and/or reduction of the motor nerve conduction velocity (MNCV) (conduction block was defined as a 50% reduction of the amplitude after proximal stimulation without significant dispersion relative to the distal (popliteal fossa) CMAP) and 3) lumbar root involvement, depicted by prolongation of F-wave latencies.

**Fig 2:**
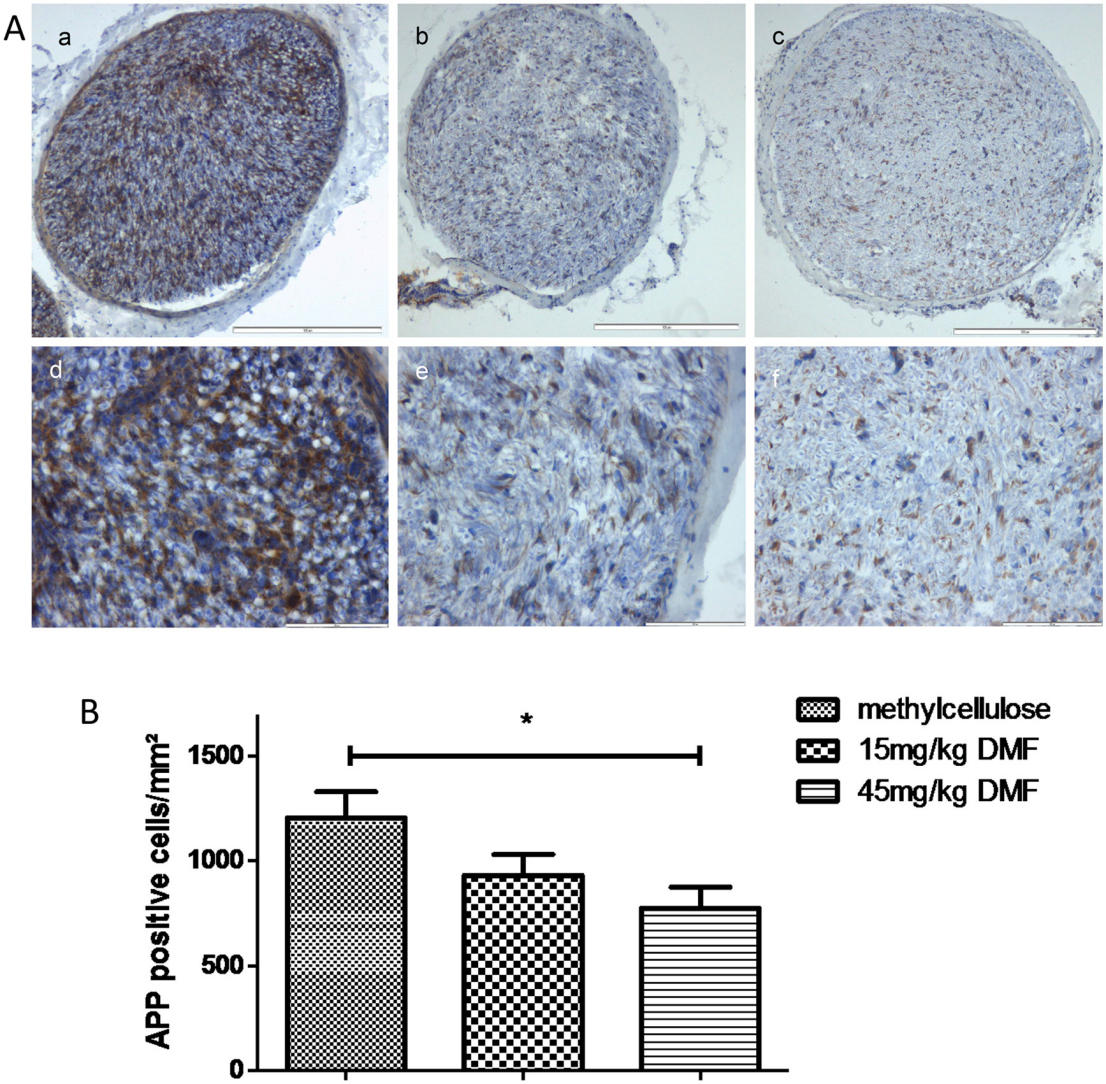
Dimethyl fumarate improved proximal and distal nerve conduction. (A) Representative CMAP (compound motor action potentials) traces during EAN course at days −1 and 16 p.i. showing a conduction block for methylcellulose-treated rats at day 16 p.i. whereas for 45 mg/kg DMF-treated rats no conduction block was recorded. (B) Representative F-wave traces after distal stimulation showing prolonged F-waves latencies only for the methylcellulose-treated group at day 16 p.i. in comparison to day -1. Rats treated with 45mg/kg did not show any significant differences in the F-wave latencies between day -1 and 16 p.i. The black vertical line defines the motor (M) response and the F (F-wave) response latency. On the left of the red vertical line applies the M response regarding distance (horizontally, ms) and vertically (mV) and on the right of the red vertical line applies the F response data (ms, mV), (M: M response, F: F response, D: distance of one side of the dotted lined squares). (C) After proximal and distal stimulation of the sciatic nerve the conduction velocity was calculated. A statistical significant reduction of the MNCV (motor nerve conduction velocity) appeared for the control group and the 15mg/kg group (p<0,0001 ***, n = 10), but no difference in the MNCV was seen for the 45mg/kg DMF treated group indicating a protective role of DMF against demyelination. Mean values and SEM are depicted.

The electrophysiological measurements of the sciatic nerve at the maximum of the clinical course (day 16 p.i.) showed a significant reduction of the MNCV in the methylcellulose-treated rats (mean MNCV on day 16 p.i. 31.9 m/s vs. day -1 p.i. 46.9 m/s, ***p<0.0001, n = 10) and the rats treated with 15 mg/kg DMF, a non-significant reduction of MNCV for the 30 mg/kg DMF treated group, whereas no difference was seen on day 16 p.i. as compared to the mean MNCV on day -1 for 45 mg/kg DMF-treated group (Fig 2).

At this time point a synchronous distal and proximal reduction of the CMAP amplitude was measured for 40% of sham-treated controls and for 40% of the 15mg/kg treated group but in no rat of the 45mg/kg treated group (n = 10), indicating a protective role of DMF against a conduction block located in the nerve roots or the peripheral nerve myelin sheath (*p<0.05). 20% of the control group animals and 20% of the 15 mg/kg DMF treated group showed a conduction block on day 16 p.i., whereas no animals receiving 45 mg/kg DMF showed a conduction block (Fig 2A).

No statistical significant differences regarding the persistence of F-wave response, as defined by the number of F-waves elicited after 10 distal stimulations or the average minimum latencies of the elicitable F-waves were observed for the DMF treated groups but prolonged F-waves were measured for the methylcellulose-treated control group at day 16 p.i., indicating that DMF reduced proximal demyelination as well (for control-group F-wave minimal latency 9.9 ms at day 16 p.i. vs 8.2 ms at day -1 p.i., *p <0,05, Fig 2B).

The MNCV was still reduced for the methylcellulose-treated rats (mean MNCV on day 23 p.i. 35.1 m/s vs. day -1 p.i. 44.3 m/s, n = 8) and for the groups treated with 15mg/kg DMF (mean MNCV on day 23 p.i. 29.4 m/s vs. day -1 p.i. 45.8 m/s, *p<0,05, n = 8) and 30mg/kg DMF, whereas no difference as compared to the mean MNCV on day -1 was seen for the 45 mg/kg DMF-treated group (mean MNCV on day 23 p.i. 52.3 m/s vs. day -1 p.i. 50.8 m/s, p>0,05, n = 8), indicating the protective role of DMF against demyelination even at this late time point with scarce clinical signs of the disease.

No significant differences regarding the persistence of F-wave response, as defined by the number of F-waves elicited after 10 distal stimulations or the average minimum latencies of the elicitable F-waves were observed at day 23 p.i. (data not shown).

### Dimethyl fumarate reduces T cell and macrophage infiltration in peripheral nerves

**Fig 3:**
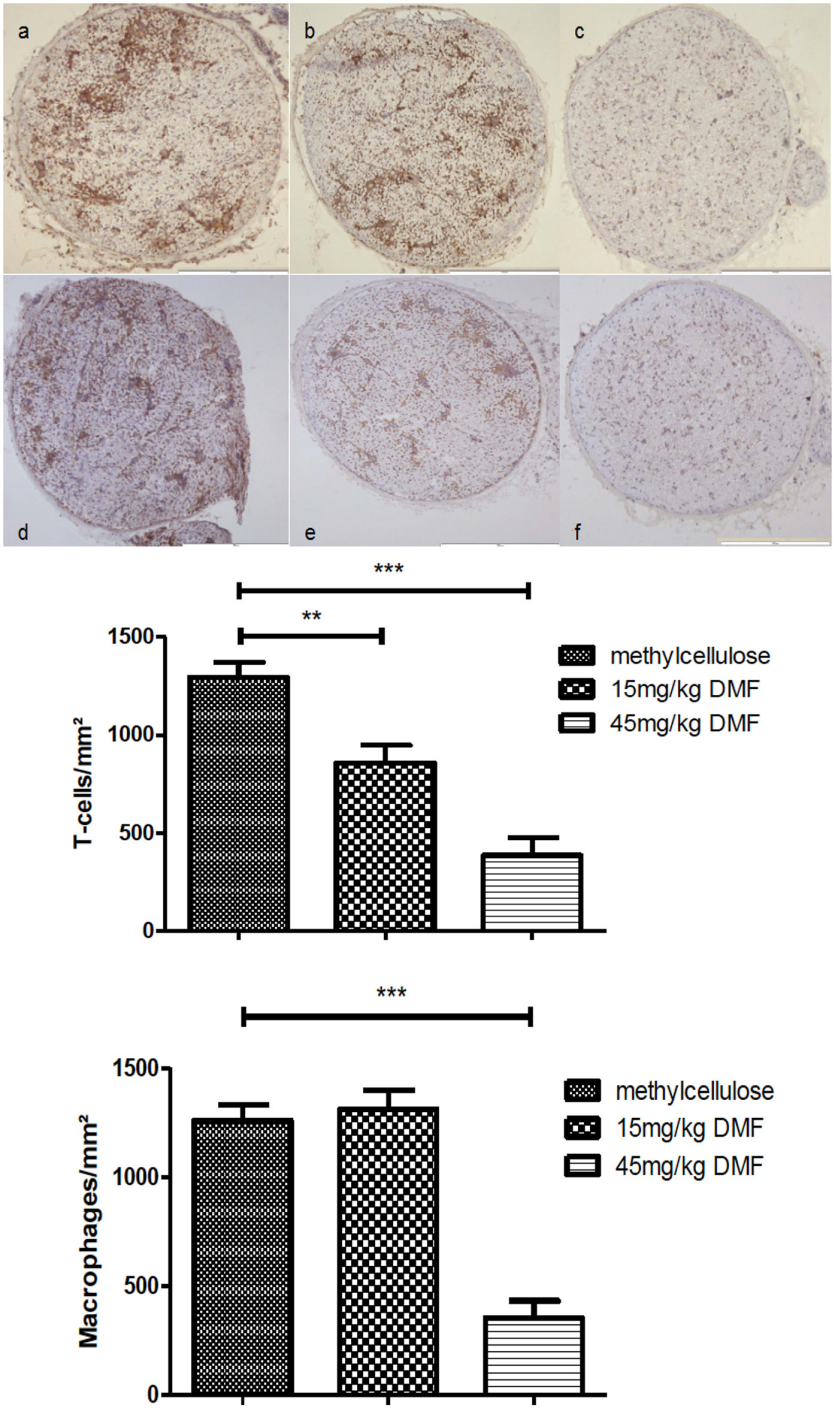
Dimethyl fumarate reduced inflammatory infiltrates of T cells and macrophages in sciatic nerves of EAN rats. (A) Rats were daily force fed with DMF or tap water and 16 days p.i. (at expected disease maximum), sciatic nerves were isolated and stained for CD3^+^ cells (a, b, c) and CD68^+^ cells (macrophages) (d, e, f). Representative photos of sciatic nerves in transverse sections of methylcellulose-treated animals (a and d), 15 mg/kg DMF-treated animals (b and e) and 45mg/kg DMF treated animals (c and f). Scale bars indicate 100μm. (B) Mean numbers of T cells per mm^2^ sciatic nerve sections and B. Mean numbers of macrophages (CD68^+^) per mm^2^ sciatic nerve sections as calculated by immunohistochemistry on day 16 p.i. from EAN rats (n = 6/group) receiving orally DMF at different doses (15mg/kg, 45mg/kg/day) and methylcellulose-treated rats. Mean values and SEM are depicted (** p<0,005, ***p<0,0001). The experiment was repeated 2 times with similar results.

We next analysed if the therapeutic efficacy of DMF correlates with a reduction of inflammatory infiltration of the PNS. Histopathological data showing inflammation within the sciatic nerves are depicted in Fig 3. Administration of 45mg/kg DMF reduced significantly the degree of macrophage- and lymphocyte- infiltration at the peak of the disease (day 16 p.i.) compared to methylcellulose-treated group (Fig 3A, 3B and 3C ***p<0.001). The groups treated with 15mg/kg DMF showed a significant reduction of T cell infiltration, yet no reduction of the macrophage infiltrates was found at this dosage (Fig 3C).

### Effects of DMF on immune cell populations in peripheral lymph nodes and spleen

Next we investigated potential immunmodulatory mechanisms for the reduction of infiltrating T cells and macrophages in the peripheral nerves during DMF treatment.

We analysed by FACS the effect of DMF on effector cells (CD4^+^ cells or macrophages) and immunregulatory cell populations in the peripheral lymphoid organs at the peak of disease (day 16 p.i., n = 6). We did not observe any change in the frequency of CD4^+^ T cells or CD11b^+^ DCs between methylcellulose-treated and DMF-treated animals in spleen or lymph nodes (p>0,05).

CD4-positive regulatory T cells (CD4^+^CD25^+^FoxP3^+^ T cells) in peripheral lymph nodes and spleen did not show any significant change between sham- and DMF-treated groups in peripheral lymphoid organs (in the lymph nodes, percentage of CD4^+^CD25^+^FoxP3^+^ cells at day 16 p.i. methylcellulose-treated group: 3.5% vs. 45mg/kg DMF-treated group: 3.3%, p>0.05, Geometric Mean (GeoMean) of FoxP3 on CD4^+^CD25^+^ cells at day 16 p.i. methylcellulose-treated group: 1760 vs. 45mg/kg DMF-treated group: 1688, p>0.05). Regarding different populations of dendritic cells (CD11b^+^CD4^+^+/-MHCII) we found no significant changes after DMF treatment.

In order to dissect potential qualitative immunmodulatory mechanisms after DMF treatment we investigated the expression of IL-10 and IL-17 cytokines on MNC of the peripheral lymphoid organs through intracellular FACS staining. Again no statistically significant differences were found (data not shown).

### Dimethyl fumarate reduces early signs of axonal degeneration

Our electrophysiological data may indicate a possible protective effect of DMF on axonal damage at the peak of disease. We therefore proceeded to confirm these results in terms of histological signs of early axonal damage and therefore stained for APP.

**Fig 4:**
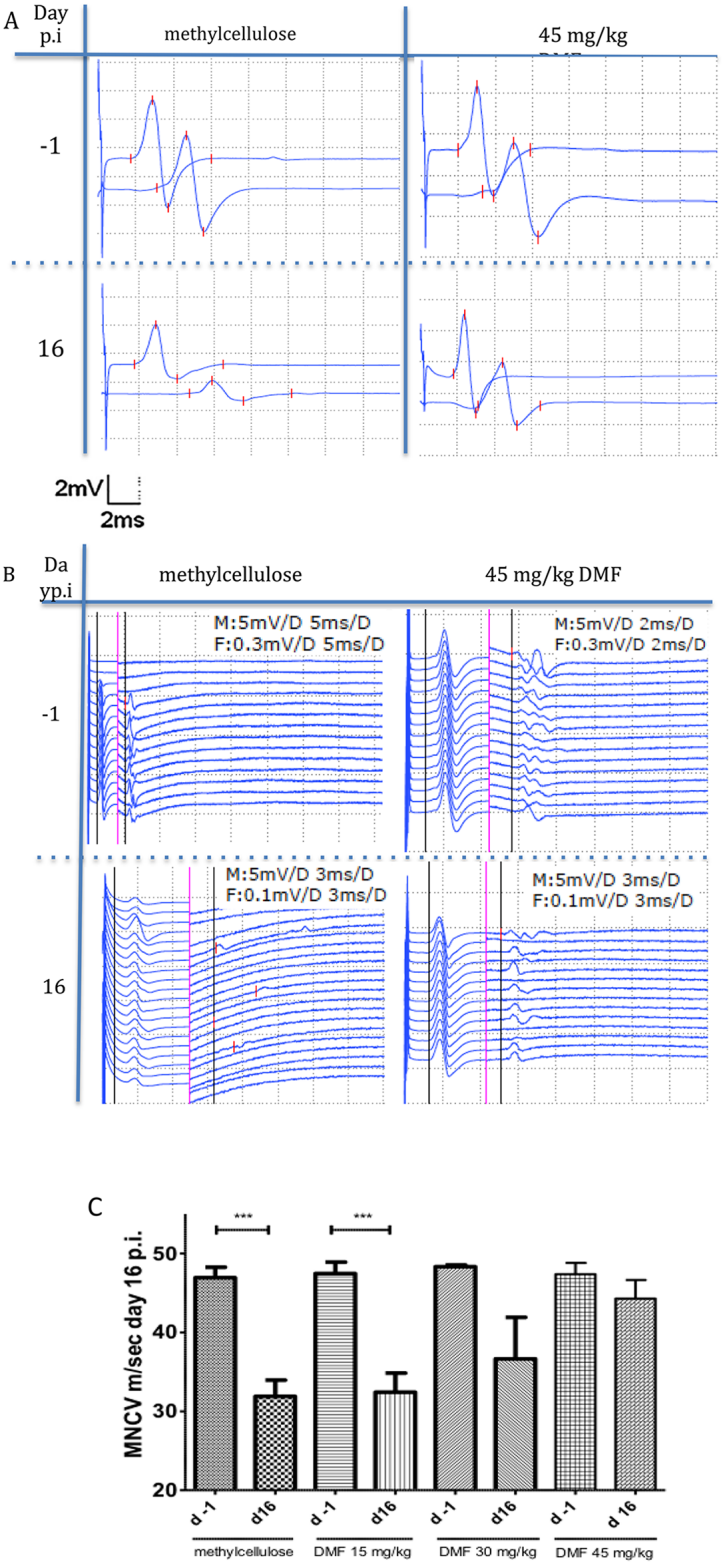
Dimethyl fumarate reduced early axonal damage at the peak of EAN course. (A) Representative photos of APP (amyloid precursor protein) staining for sciatic nerve transverse sections of rats (n = 6/group) treated with DMF 15mg/kg (b, e), 45mg/kg (c, f) and methylcellulose-treated animals (a, d), showing an reduction of APP positive cells for DMF-treated rats. Scale bars indicate 100μm for a-c and 50μm for d-f. (B) Mean numbers of APP positive cells per mm^2^ sciatic nerve sections as calculated by immunohistochemistry on day 16 p.i. from EAN rats (n = 6/group) receiving orally DMF at different doses (15mg/kg, 45mg/kg/day) and methylcellulose-treated rats. Mean values and SEM are depicted (*p<0,05). The experiment was repeated 2 times with similar results.

Histological data showing APP staining within the sciatic nerves are depicted in Fig 4A. Administration of 45mg/kg DMF reduced significantly APP positive axons at the peak of the disease (day 16 p.i.) compared to methylcellulose-treated group (Fig 4B *p<0.05, n = 6). The groups treated with 15mg/kg DMF showed no significant reduction of APP positive axons.

### Dimethyl fumarate induces Nrf2 in axons at the peak of clinical EAN course

We next proceeded to investigate potential mechanisms for this neuroprotective effect, which have been suggested in previous EAE studies [15].

**Fig 5:**
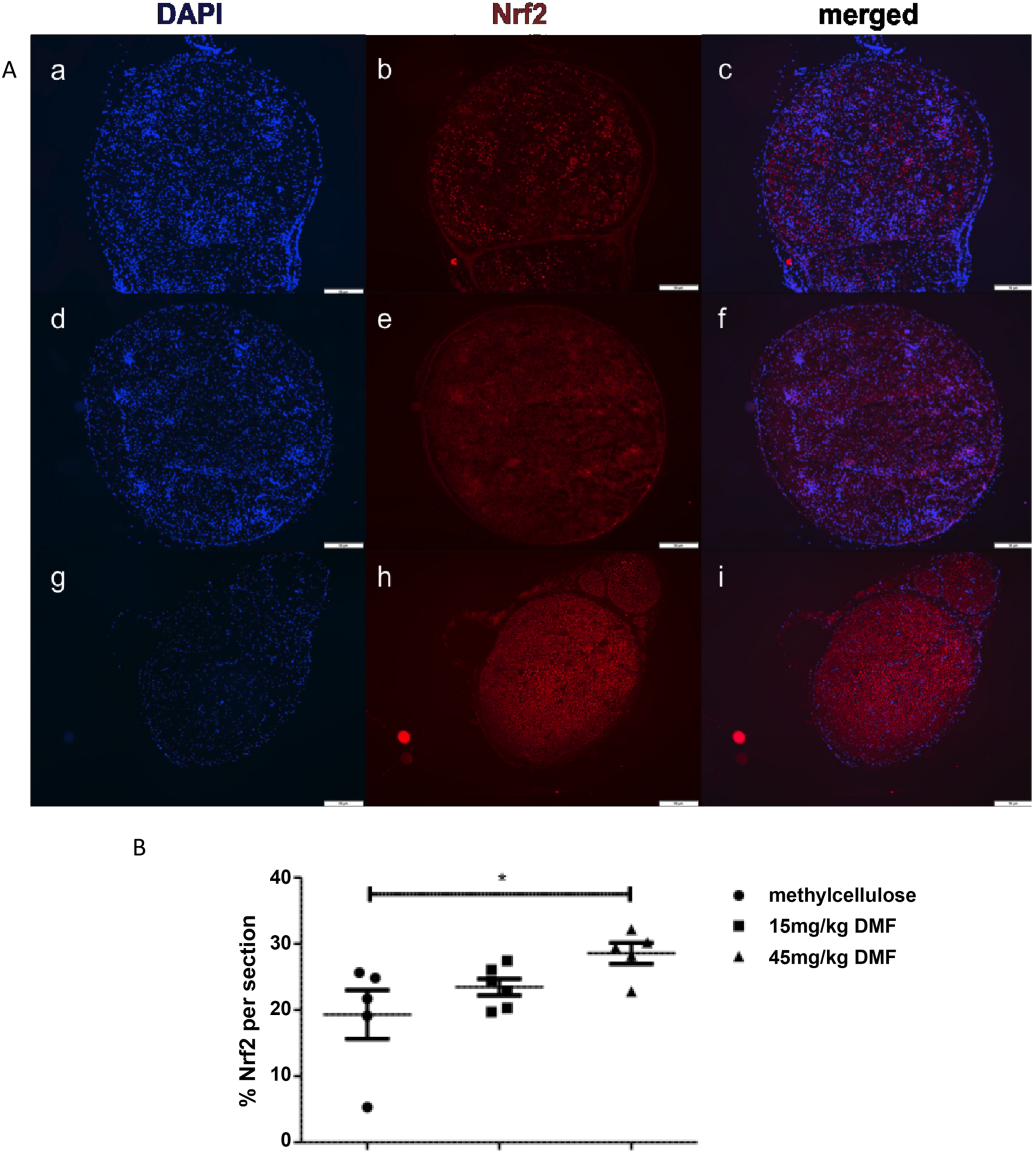
Dimethyl fumarate induced Nrf2 at the peak of EAN course. (A) Representative photos of Nrf2 staining for sciatic nerve transverse sections of rats (n = 6/group) treated with DMF 15mg/kg (d-f), 45mg/kg (g-i) and methylcellulose-treated animals (a-c), showing an increase of Nrf2 positive cells for 45mg/kg DMF-treated rats. Pictures a, d and g show nuclear stain (DAPI), pictures b, e and h Nrf2 stain and pictures c, f and i indicate double staining. Scale bars indicate 100μm. (B) Percentage of Nrf2 positive staining per sciatic nerve section measured by immunofluorescent staining on day 16 p.i. from EAN rats (n = 6/group) receiving DMF at different doses (15mg/kg, 45mg/kg/day) and methylcellulose-treated rats. Mean values and SEM are depicted (*p<0,05).

Representative pictures from an immunofluorescent staining, showing Nrf2 positive cells in the sciatic nerves are depicted in Fig 5 and revealed a significantly increased expression of Nrf2 in the peripheral nerves of 45mg/kg DMF treated group at the peak of disease (*p<0,05, n = 6).

**Fig 6:**
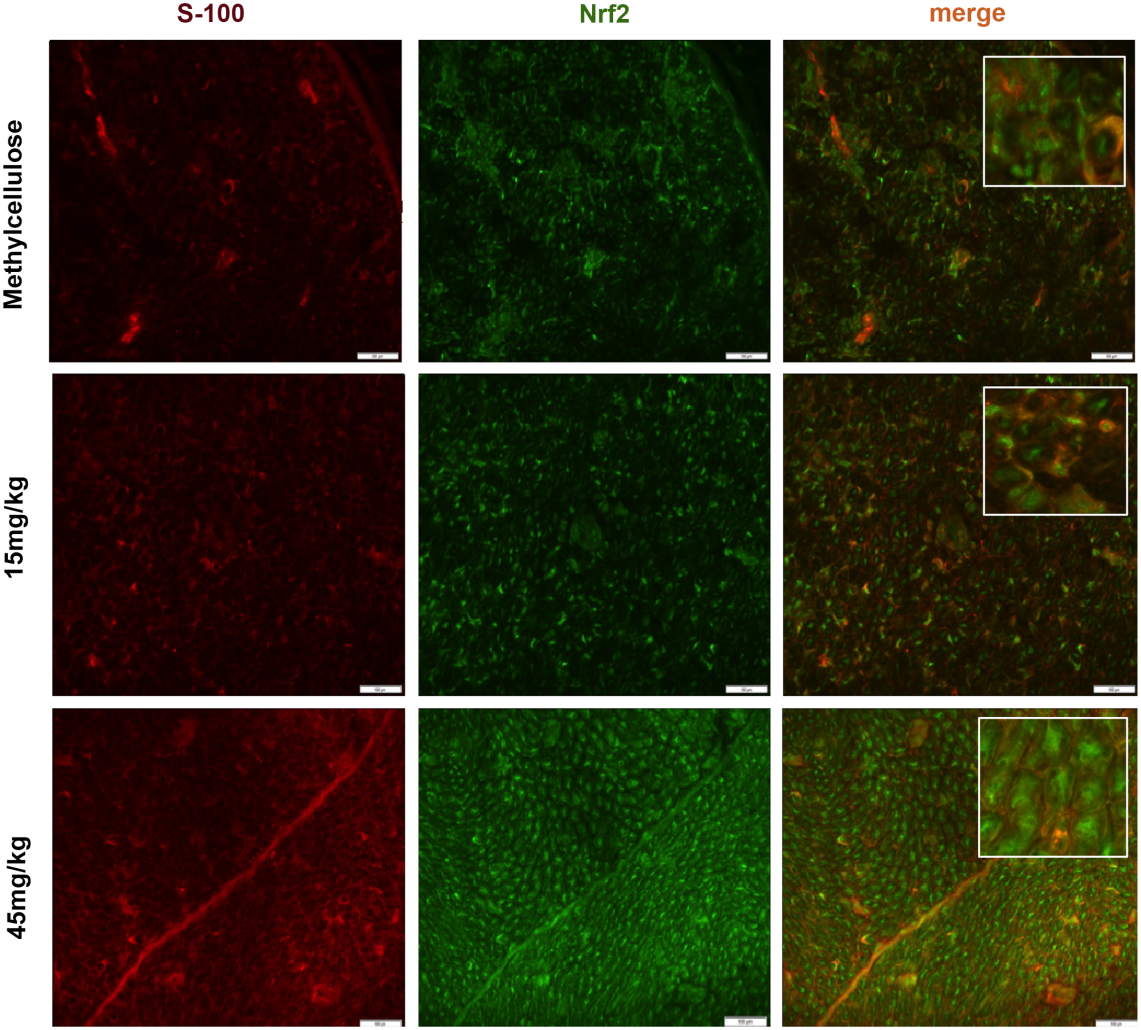
Dimethyl fumarate did not induce Nrf2 in Schwann cells at the peak of EAN course. Representative photos of double (merge) Nrf2 positive Schwann cells (S100 positive) staining for sciatic nerve transverse sections of rats (n = 6/group) treated with DMF 15mg/kg, 45mg/kg and methylcellulose. No statistical significant increase between Nrf2 positive Schwann cells for DMF-treated vs. methylcellulose-treated rats on day 16 p.i. was detected (insets depict details of staining). Scale bars indicate 50μm.

**Fig 7:**
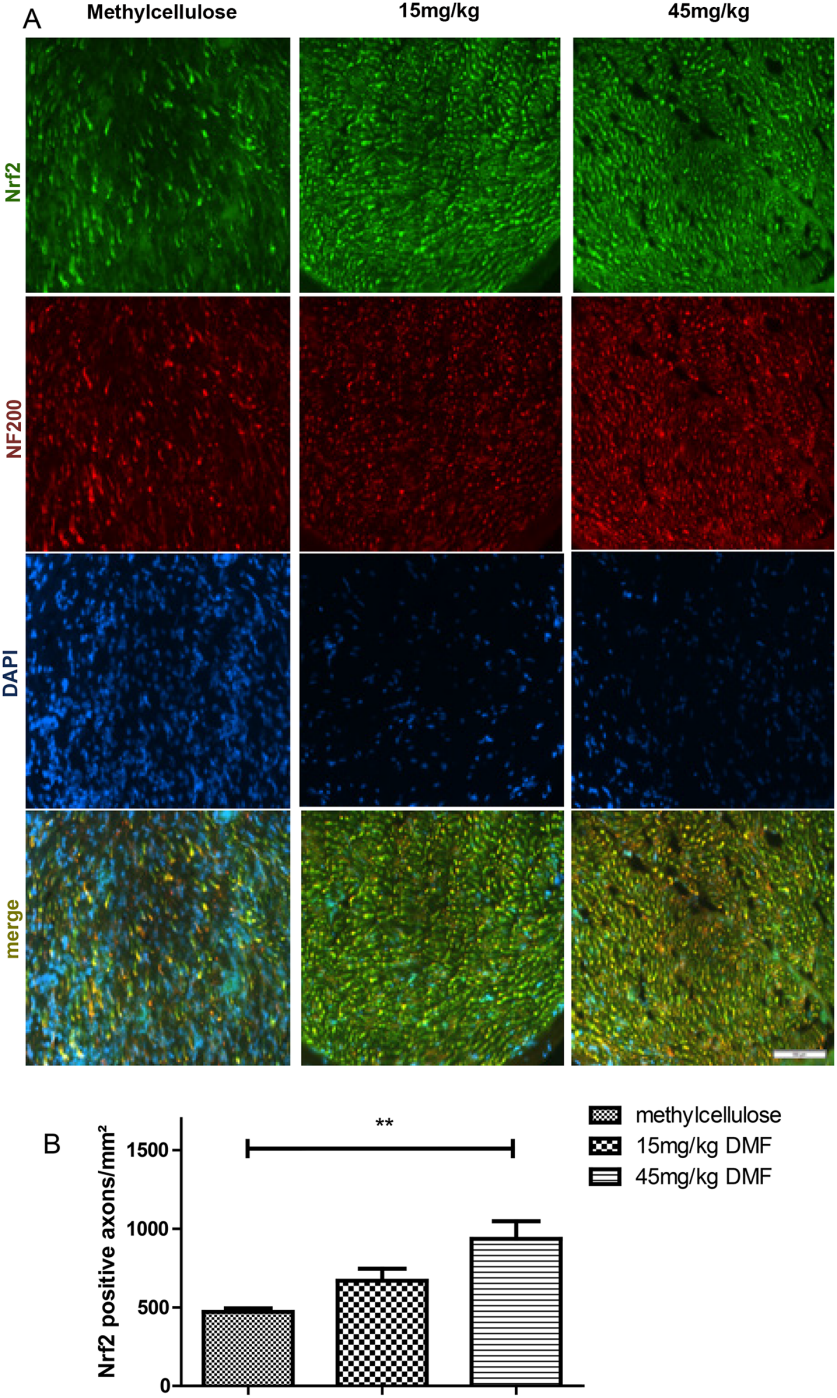
Dimethyl fumarate induced Nrf2 in axons at the peak of EAN course. (A) Representative photos of double (merge) Nrf2-NF (neurofilament-axons) staining for sciatic nerve transverse sections of rats (n = 6/group) treated with DMF 15mg/kg, 45mg/kg and methylcellulose, showing Nrf2 positive axons. Scale bar indicates 50μm. (B) Mean numbers of Nrf2 positive axons per mm^2^ sciatic nerve sections as calculated by immunohistochemistry on day 16 p.i. from EAN rats (n = 6/group) receiving orally DMF at different doses (15mg/kg, 45mg/kg/day) and methylcellulose-treated rats. Mean values and SEM are depicted (**p<0,005). The experiment was repeated 2 times with similar results.

In order to investigate the structures expressing Nrf2 in the peripheral nerves we performed double staining for Nrf2-S100 (Fig 6) and for Nrf2-NF200 (Fig 7A), which revealed that axons and not Schwann cells showed an induction of Nrf2 after dimethyl fumarate treatment on day 16 p.i. (Fig 7B, **p<0.05). A possible protective role of DMF on Schwann cells was investigated with a double staining TUNEL-S-100 staining, which did not show any significant reduction of Schwann cell death in the DMF treated groups (data not shown).

## Discussion

In the present study we investigated the therapeutic effects of orally administered dimethyl fumarate in EAN of Lewis rats. We were able to show its preventive therapeutic efficacy in EAN resulting in amelioration of the disease course. This went along with reduced T cell and macrophage inflammation, reduced demyelination and higher survival of axons as reflected by electrophysiology and histology.

Interestingly, only one concentration of dimethyl fumarate (45 mg/kg) showed a robust and significant effect in the treatment of EAN, with an improvement of the clinical score, inflammatory and electrophysiological parameters. This was in contrast with previous EAE studies in mice, where the concentrations of 15 mg/kg exhibited a marked preventive effect [27]. This discrepancy may be attributed to the fact that the concentration of 15 mg/kg DMF induced only a reduction of T cells and failed to induce a reduction of CD68^+^ macrophages in rat peripheral nerves, which represent a main inflammatory population in EAN. Also strain specific differences in metabolism may exist between mice and rats. Toxic effects were observed neither with 45 mg/kg DMF nor with 100 mg/kg DMF. These findings raise the necessity of a careful titration of dimethyl fumarate for each species in order to optimize its therapeutic effects.

Taking advantage of the easy accessibility of the PNS for nerve conduction studies, we found a significant reduction of electrophysiological signs of demyelination for the 45 mg/kg DMF groups both in proximal (increase of F-wave latencies) and distal parts (reduction of motor nerve conduction) of the sciatic nerve. Protection against demyelination correlated with a reduction of T cells and macrophages in the PNS.

We then evaluated potential immunological mechanisms responsible for this reduction of immune infiltration of the peripheral nerves, which of course may also indirectly support survival of axonal structures. Regarding effector cells we found no significant modulation neither of the percentage of CD4^+^ and CD11b^+^ cells nor of regulatory populations (regulatory T cells, dendritic cells) in the spleen or lymph nodes of DMF treated rats. There was also no difference between the production of IL-10 and IL-17 from MNC in the peripheral lymphoid organs.

Our experiments could not confirm the reports from in vitro experiments in murine EAE, which have shown that DMF induced T helper 2 cells and generated type II dendritic cells (DCs) that produce IL-10 [28,29,30]. However our findings are in line with the findings of Schilling et al, who could not confirm these effects in murine EAE in vivo [14]. Again it may be of note that even in mice different immune effects were seen in the SJL strain [29] but not C57/BL6 strain [14]. On the other hand, we have to point out that the effector and regulatory cell populations were examined at the maximum of clinical disease (day 16 p.i.). A regulation of the immune system through DMF could also precede the reduction of immune infiltrates in the peripheral nerves and may have occurred early at the beginning of the disease (day 11 p.i.).

Beyond peripheral immunmodulatory mechanisms, the reduction of inflammatory infiltrates in EAN may be attributed to the interference of dimethyl fumarate with the adhesion of effector cells to endothelial cells and reduction of chemotaxis [31].

Axonal damage already in early disease stages is a crucial pathophysiological aspect of autoimmune diseases of the central as well as of the peripheral nervous system. Long-term disability in acute and chronic inflammatory demyelinating neuropathy is crucially determined by the degree of axonal degeneration [32]. There are questions as to whether the release of neurotoxic cytokines (e.g., tumor necrosis factor a) and noxious mediators (e.g., nitric oxide and metalloproteinases) enhance axonal destruction, but it has become clear that early, effective therapy minimizes axonal loss [6]. Therapeutic substances with neuroprotective potential for peripheral autoimmune neuropathies are as yet not approved.

In our model, although primarily representing a demyelinating neuropathy, we found signs of early axonal degeneration in nerve conduction studies and histology, which were attenuated for the 45 mg/kg DMF treated group. These findings correlated with a statistical significant increase of Nrf2 positive structures in the sciatic nerves as shown by immunohistochemistry. A histological analysis of different cells populations expressing Nrf2 revealed that it was primarily expressed in axons and not in Schwann cells, which have been implicated as active participants in autoimmune inflammation and neuroprotection [33,34].

Our findings are in line with previous publications of our group in EAE suggesting that fumarates activate the Nrf2 transcriptional pathway in neurons. They showed in chronic EAE that DMF modifies cysteine 151 on inhibitor Keap1, thereby activating Nrf2, which binds to antioxidant response elements in the promoters of protective genes such as NADPH-quinone-oxidoreductase-1 (NQO1) [15] and heme-oxygenase-1 [17]. This ultimately raises the levels of the important intracellular antioxidant glutathione exerting neuroprotective effects. Studies in Nrf2-knockout mice revealed that most of the therapeutic ability of fumarates was abolished in the absence of Nrf2 [15]. In acute EAE Reick *et al*. also reported an increased axonal density on day 18 p.i. after DMF treatment [27].

In conclusion, the clinical severity of EAN and inflammatory nerve infiltrates are markedly suppressed by oral treatment with dimethyl fumarate. In addition upregulation of Nrf-2 and improved axonal survival is observed. Dimethyl fumarate may therefore be an attractive candidate for the treatment of autoimmune diseases of the PNS, and may open neuroprotective avenues for this devastating group of diseases.

## Supporting Information

S1 Fig Clinical EAN course under dimethyl fumarate treatment. Analytical data depicting the clinical course and weight changes during EAN induced in Lewis rats by immunisation on day 0 with P2 peptide 53–78 plus CFA. Rats received DMF diluted in 0,08% methylcellulose in tap water at doses of 15 mg/kg, 30mg/kg and 45mg/kg twice daily from day 0 to day 23-post immunisation by oral gavage. Control rats received 0,08% methylcellulose in tap water only. Mean values and SEM are depicted, ROC Area under curve (AUC) 45mg/kg vs. methylcellulose, n = 8 * p<0,05. The experiment was repeated 2 times with similar results.
(PZF)

S2 Fig Dimethyl fumarate improved proximal and distal nerve conduction. After proximal and distal stimulation of the sciatic nerve the conduction velocity was calculated. Analytical data providing the calculations are provided. A statistical significant reduction of the MNCV (motor nerve conduction velocity) appeared for the control group and the 15mg/kg group (p<0,0001 ***, n = 10), but no difference in the MNCV was seen for the 45mg/kg DMF treated group on day 16 p.i. indicating a protective role of DMF against demyelination. Mean values and SEM are depicted. The F waves of all groups calculated at the end of the disease showed no statistical significant increase.
(PZF)

S3 Fig Dimethyl fumarate reduced inflammatory infiltrates of T cells in sciatic nerves of EAN rats. Analytical data and mean numbers of T cells per mm^2^ sciatic nerve sections and as calculated by immunohistochemistry on day 16 p.i. from EAN rats (n = 6/group) receiving orally DMF at different doses (15mg/kg, 45mg/kg/day) and methylcellulose-treated rats. Mean values and SEM are depicted (** p<0,005, ***p<0,0001). The experiment was repeated 2 times with similar results.
(PZF)

S4 Fig Dimethyl fumarate reduced inflammatory infiltrates of macrophages in sciatic nerves of EAN rats. Analytical data and mean numbers of macrophages per mm^2^ sciatic nerve sections and as calculated by immunohistochemistry on day 16 p.i. from EAN rats (n = 6/group) receiving orally DMF at different doses (15mg/kg, 45mg/kg/day) and methylcellulose-treated rats. Mean values and SEM are depicted (** p<0,005, ***p<0,0001). The experiment was repeated 2 times with similar results.
(PZF)

S5 Fig Dimethyl fumarate reduced early axonal damage at the peak of EAN course. Analytical data and mean numbers of APP positive cells per mm^2^ sciatic nerve sections as calculated by immunohistochemistry on day 16 p.i. from EAN rats (n = 6/group) receiving orally DMF at different doses (15mg/kg, 45mg/kg/day) and methylcellulose-treated rats. Mean values and SEM are depicted (*p<0,05). The experiment was repeated 2 times with similar results.
(PZF)

S6 Fig Dimethyl fumarate induced Nrf2 at the peak of EAN course. Analytical data and mean values of the percentage of Nrf2 positive staining per sciatic nerve section measured by immunofluorescent staining on day 16 p.i. from EAN rats (n = 6/group) receiving DMF at different doses (15mg/kg, 45mg/kg/day) and methylcellulose-treated rats. Mean values and SEM are depicted (*p<0,05).
(PZF)

S7 Fig Dimethyl fumarate induced Nrf2 in axons at the peak of EAN course. Analytical data and mean numbers of Nrf2 positive axons per mm^2^ sciatic nerve sections as calculated by immunohistochemistry on day 16 p.i. from EAN rats (n = 6/group) receiving orally DMF at different doses (15mg/kg, 45mg/kg/day) and methylcellulose-treated rats. Mean values and SEM are depicted (**p<0,005). The experiment was repeated 2 times with similar results.
(PZF)

## Abbreviations

APP: Amyloid precursor protein
CFA: Complete Freund´s Adjuvant
CIDP: Chronic inflammatory demyelinating polyneuropathy
CMAP: Compound motor action potential
DC: Dendritic cells
DMF: Dimethyl fumarate
EAE: Experimental autoimmune encephalomyelitis
EAN: Experimental autoimmune neuritis
FACS: Fluorescent associated cell sorting
GBS: Guillain-Barré Syndrome
i.p.: intraperitoneally
MMF: Monomethyl fumarate
MNC: mononuclear cells
MNCV: Motor nerve conduction velocity
Nrf-2: nuclear factor (erythroid derived 2)-related factor 2
p.i.: post immunisation
PNS: Peripheral nervous system

## References

[1] Yoon MS, Chan A, Gold R. Standard and escalating treatment of chronic inflammatory demyelinating polyradiculoneuropathy. Ther Adv Neurol Disord. 2011 5;4(3):193–200. 10.1177/1756285611405564 21694819 PMC3105635

[2] Hughes RA, Pritchard J, Hadden RD. Pharmacological treatment other than corticosteroids, intravenous immunoglobulin and plasma exchange for Guillain-Barré syndrome. Cochrane Database Syst Rev. 2013. 10.1002/14651858.CD008630.pub3 23450584

[3] Zhang HL, Zheng XY, Zhu J. Th1/Th2/Th17/Treg cytokines in Guillain-Barré syndrome and experimental autoimmune neuritis. Cytokine Growth Factor Rev. 2013 10;24(5):443–53. 10.1016/j.cytogfr.2013.05.005 23791985

[4] Hughes RA. Management of chronic inflammatory demyelinating polyradiculoneuropathy. Drugs. 2003;63(3):275–87. Review. 12534332 10.2165/00003495-200363030-00003

[5] Kieseier BC, Kiefer R, Gold R, Hemmer B, Willison HJ, Hartung HP. Advances in understanding and treatment of immune-mediated disorders of the peripheral nervous system. Muscle Nerve. 2004 8;30(2):131–56. Review. 15266629 10.1002/mus.20076

[6] Mathey EK, Park SB, Hughes RA, Pollard JD, Armati PJ, Barnett MH, et al Chronic inflammatory demyelinating polyradiculoneuropathy: from pathology to phenotype. J Neurol Neurosurg Psychiatry. 2015 2 12 pii: jnnp-2014-309697. 10.1136/jnnp-2014-309697 [Epub ahead of print] Review. PMC4552934 25677463

[7] Waksman BH, Adams RD. Allergic neuritis: an experimental disease of rabbits induced by the injection of peripheral nervous tissue and adjuvants. J Exp Med. 1955 8 1;102(2):213–36. 13242745 10.1084/jem.102.2.213 PMC2136504

[8] Kadlubowski M, Hughes RA. Identification of the neuritogen for experimental allergic neuritis. Nature. 1979 1 11;277(5692):140–1. 310522 10.1038/277140a0

[9] Kiefer R, Kieseier BC, Stoll G, Hartung HP. The role of macrophages in immune- mediated damage to the peripheral nervous system. Prog Neurobiol 2001;64:109–27. 65. 11240209 10.1016/s0301-0082(00)00060-5

[10] Kieseier BC, Dalakas MC, Hartung HP. Immune mechanisms in chronic inflammatory demyelinating neuropathy. Neurology. 2002 12 24;59(12 Suppl 6):S7–12. Review. 12499465 10.1212/wnl.59.12_suppl_6.s7

[11] Gold R. Experimental autoimmune neuritis In: Dyck PJ, Thomas PK, editors, Peripheral Neuropathy, Saunders, 4th Edition, 2005)

[12] Gold R, Kappos L, Arnold DL, Bar-Or A, Giovannoni G, Selmaj K, DEFINE Study Investigators et al Placebo-controlled phase 3 study of oral BG-12 for relapsing multiple sclerosis. N Engl J Med. 2012;367(12):1098–1107. 22992073 10.1056/NEJMoa1114287

[13] Fox RJ, Miller DH, Phillips JT, Hutchinson M, Havrdova E, Kita M, CONFIRM Study Investigators et al Placebo-controlled phase 3 study of oral BG-12 or glatiramer in multiple sclerosis. N Engl J Med. 2012;367(12):1087–1097. 22992072 10.1056/NEJMoa1206328

[14] Schilling S, Goelz S, Linker RA, Luehder F, Gold R. Fumaric acid esters are effective in chronic experimental autoimmune encephalomyelitis and suppress macrophage infiltration, Clin. Exp. Immunol. 145 (2006) 101–107. 16792679 10.1111/j.1365-2249.2006.03094.x PMC1942010

[15] Linker RA, Lee DH, Ryan S, van Dam AM, Conrad R, Bista P, et al Fumaric acid esters exert neuroprotective effects in neuroinflammation via activation of the Nrf2 antioxidant pathway. Brain. 2011 3;134(Pt 3):678–92. 10.1093/brain/awq386 21354971

[16] Stoof TJ, Flier J, Sampat S, Nieboer C, Tensen CP, Boorsma BM. The antipsoriatic drug dimethylfumarate strongly suppresses chemokine production in human keratinocytes and peripheral blood mononuclear cells, Br. J. Dermatol. 144 (2001) 1114–1120. 11422029 10.1046/j.1365-2133.2001.04220.x

[17] Mrowietz U, Asadullah K. Dimethylfumarate for psoriasis: more than a dietary curiosity, Trends Mol. Med. 11 (2005) 43–48. 15649822 10.1016/j.molmed.2004.11.003

[18] Wilms H, Sievers J, Rickert U, Rostami-Yazdi M, Mrowietz U, Lucius R. Dimethylfumarate inhibits microglial and astrocytic inflammation by suppressing the synthesis of nitric oxide, IL-1beta, TNF-alpha and IL-6 in an in-vitro model of brain inflammation. J Neuroinflammation. 2010 5 19;7:30 10.1186/1742-2094-7-30 20482831 PMC2880998

[19] Bénardais K, Pul R, Singh V, Skripuletz T, Lee DH, Linker RA et al Effects of fumaric acid esters on blood-brain barrier tight junction proteins. Neurosci Lett. 2013 10 25;555:165–70. 10.1016/j.neulet.2013.09.038 24076006

[20] Altmeyer P, Hartwig R, Matthes U. Efficacy and safety profile of fumaric acid esters in oral long-term therapy with severe treatment refractory psoriasis vulgaris. A study of 83 patients, Hautarzt 47 (1996) 190–196. 8647701 10.1007/s001050050401

[21] Treumer F, Zhu K, Glaser R, Mrowietz U. Dimethylfumarate is a potent inducer of apoptosis in human T cells, J. Invest. Dermatol. 121 (2003) 1383–1388. 14675187 10.1111/j.1523-1747.2003.12605.x

[22] Nave KA, Werner HB. Myelination of the nervous system: mechanisms and functions. Annu Rev Cell Dev Biol. 2014;30:503–33. 10.1146/annurev-cellbio-100913-013101 25288117

[23] Enders U, Lobb R, Pepinsky RB, Hartung HP, Toyka KV, Gold R. The role of the very late antigen-4 and its counterligand vascular cell adhesion molecule-1 in the pathogenesis of experimental autoimmune neuritis of the Lewis rat. Brain. 1998 7;121 (Pt 7):1257–66. 9679778 10.1093/brain/121.7.1257

[24] Tuck RR, Antony JH, McLeod JG. F-wave in experimental allergic neuritis. J Neurol Sci. 1982 11;56(2–3):173–84. 7175545 10.1016/0022-510x(82)90140-x

[25] Hartung HP, Schäfer B, Heininger K, Toyka KV. Suppression of experimental autoimmune neuritis by the oxygen radical scavengers superoxide dismutase and catalase. Ann Neurol. 1988 5;23(5):453–60. 3260463 10.1002/ana.410230505

[26] Taylor JM, Pollard JD. Neurophysiological changes in demyelinating and axonal forms of acute experimental autoimmune neuritis in the Lewis rat. Muscle Nerve. 2003 9;28(3):344–52. 12929195 10.1002/mus.10432

[27] Reick C, Ellrichmann G, Thöne J, Scannevin RH, Saft C, Linker RA, et al Neuroprotective dimethyl fumarate synergizes with immunomodulatory interferon beta to provide enhanced axon protection in autoimmune neuroinflammation. Exp Neurol. 2014 7;257:50–6. 10.1016/j.expneurol.2014.04.003 24731948

[28] Zhu K, Mrowietz U. Inhibition of dendritic cell differentiation by fumaric acid esters, J. Invest. Dermatol. 116 (2001) 203–208. 11179994 10.1046/j.1523-1747.2001.01159.x

[29] Ghoreschi K, Brück J, Kellerer C, Deng C, Peng H, Rothfuss O, et al Fumarates improve psoriasis and multiple sclerosis by inducing type II dendritic cells. J Exp Med. 2011 10 24;208(11):2291–303. 10.1084/jem.20100977 21987655 PMC3201195

[30] De Jong R, Bezemer AC, Zomerdijk TP, Pouw-Kraan T, Ottenhoff TH, Nibbering PH. Selective stimulation of T helper 2 cytokine responses by the anti-psoriasis agent monomethylfumarate, Eur. J. Immunol. 26 (1996) 2067–2074. 8814248 10.1002/eji.1830260916

[31] Chen H, Assmann JC, Krenz A, Rahman M, Grimm M, Karsten CM, et al Hydroxycarboxylic acid receptor 2 mediates dimethyl fumarate's protective effect in EAE. J Clin Invest. 2014 5 1;124(5):2188–92. 24691444 10.1172/JCI72151 PMC4001545

[32] Hadden RD, Hughes RA. Management of inflammatory neuropathies. J Neurol Neurosurg Psychiatry. 2003 6;74 Suppl 2:ii9–ii14. 12754323 10.1136/jnnp.74.suppl_2.ii9 PMC1765628

[33] Meyer zu Hörste G, Hu W, Hartung HP, Lehmann HC, Kieseier BC. The immunocompetence of Schwann cells. Muscle Nerve. 2008 1;37(1):3–13. Review. 17823955 10.1002/mus.20893

[34] Pollard JD, Armati PJ. CIDP—the relevance of recent advances in Schwann cell/axonal neurobiology. J Peripher Nerv Syst. 2011 3;16(1):15–23. 10.1111/j.1529-8027.2011.00323.x 21504498

