# Correction: Dimethyl Fumarate Ameliorates Lewis Rat Experimental Autoimmune Neuritis and Mediates Axonal Protection

**DOI:** 10.1371/journal.pone.0148046

**Published:** 2016-01-25

**Authors:** The PLOS ONE Staff

Fig 2 and Fig 4 are swapped. The image in Fig 2 should be the image in Fig 4 and the image in Fig 4 should be the image in Fig 2. The captions remain unchanged. The publisher apologizes for the error. Please see the corrected Fig 2 and Fig 4 here.

**Fig 2:**
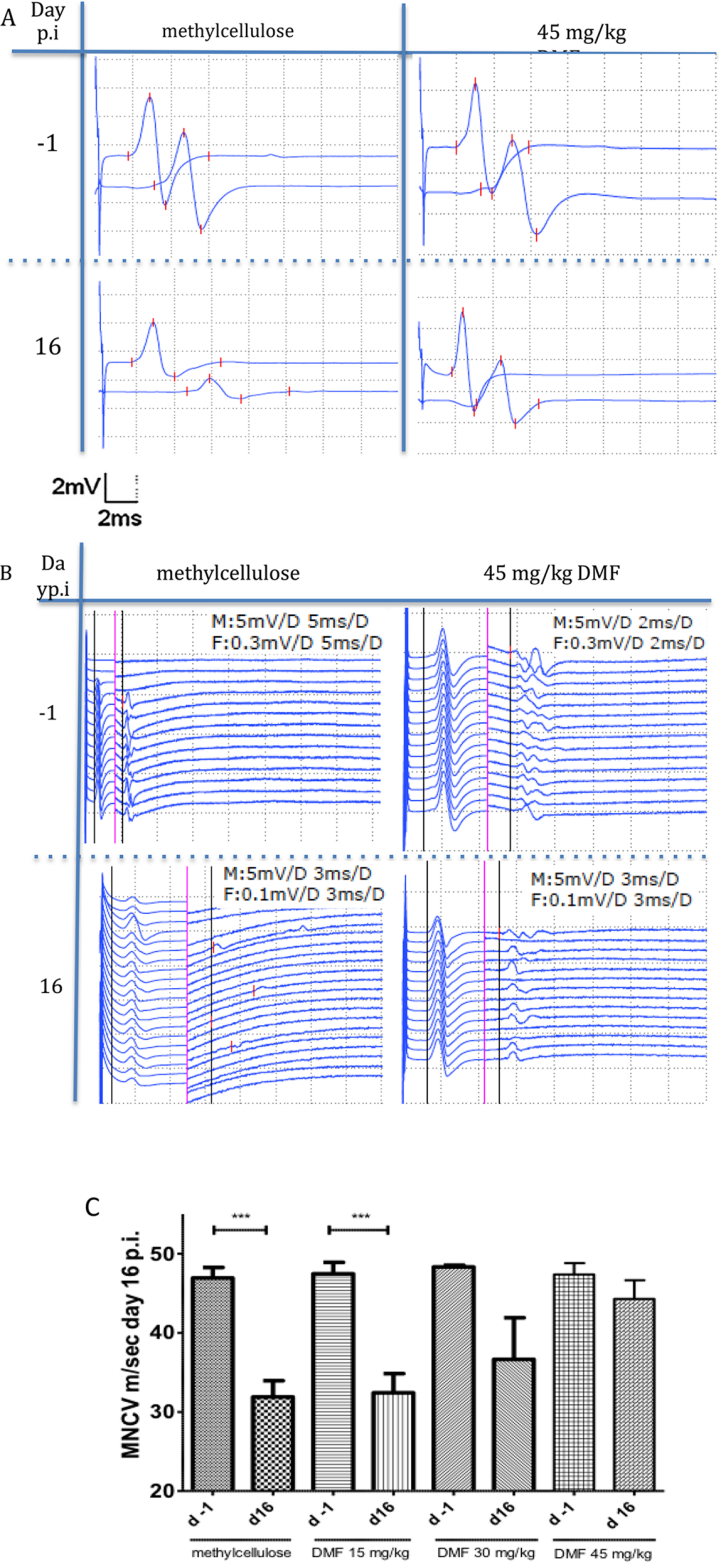
Dimethyl fumarate improved proximal and distal nerve conduction. (A) Representative CMAP (compound motor action potentials) traces during EAN course at days −1 and 16 p.i. showing a conduction block for methylcellulose-treated rats at day 16 p.i. whereas for 45 mg/kg DMF-treated rats no conduction block was recorded. (B) Representative F-wave traces after distal stimulation showing prolonged F-waves latencies only for the methylcellulose-treated group at day 16 p.i. in comparison to day -1. Rats treated with 45mg/kg did not show any significant differences in the F-wave latencies between day -1 and 16 p.i. The black vertical line defines the motor (M) response and the F (F-wave) response latency. On the left of the red vertical line applies the M response regarding distance (horizontally, ms) and vertically (mV) and on the right of the red vertical line applies the F response data (ms, mV), (M: M response, F: F response, D: distance of one side of the dotted lined squares). (C) After proximal and distal stimulation of the sciatic nerve the conduction velocity was calculated. A statistical significant reduction of the MNCV (motor nerve conduction velocity) appeared for the control group and the 15mg/kg group (p<0,0001 ***, n = 10), but no difference in the MNCV was seen for the 45mg/kg DMF treated group indicating a protective role of DMF against demyelination. Mean values and SEM are depicted.

**Fig 4:**
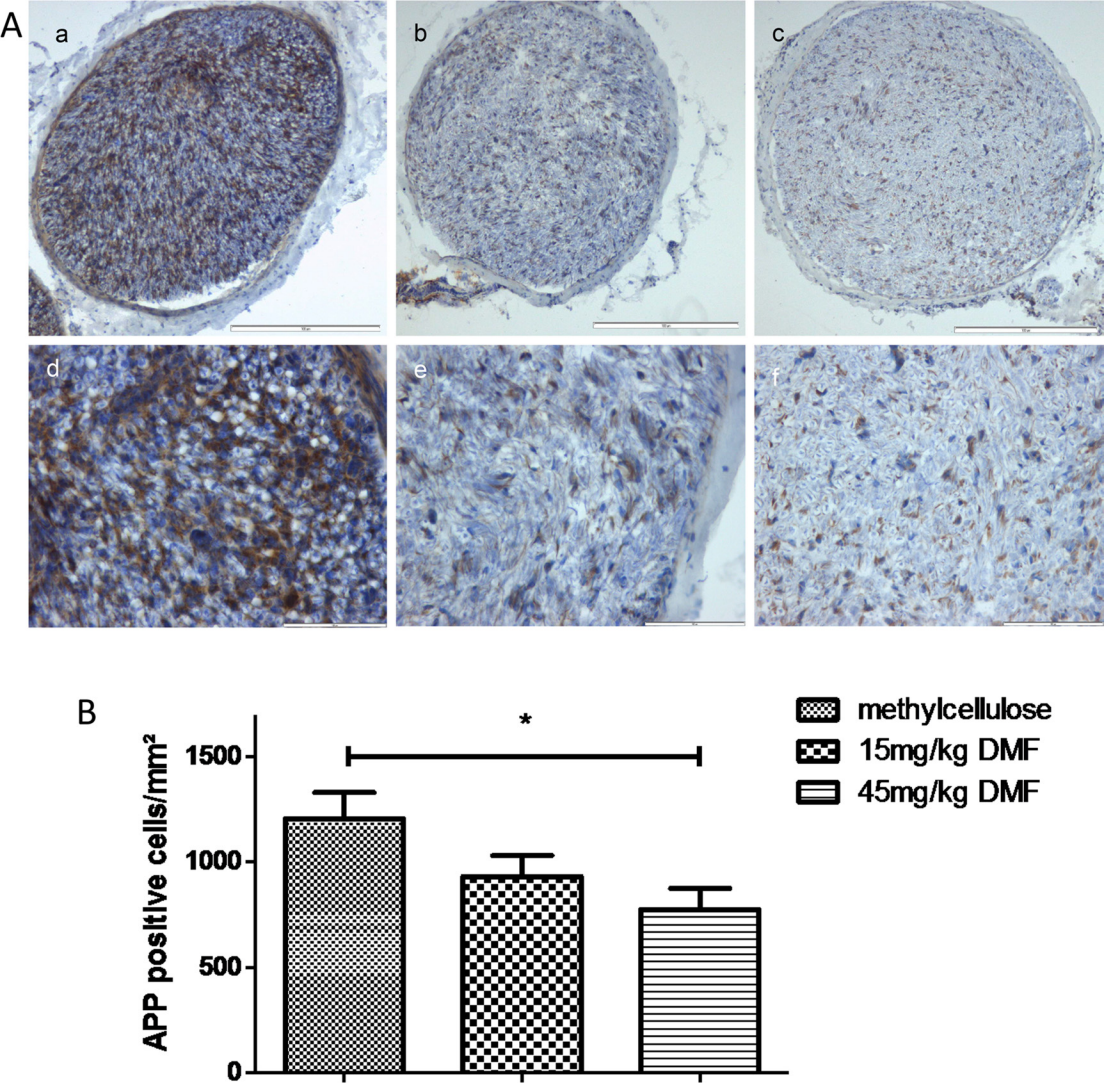
Dimethyl fumarate reduced early axonal damage at the peak of EAN course. (A) Representative photos of APP (amyloid precursor protein) staining for sciatic nerve transverse sections of rats (n = 6/group) treated with DMF 15mg/kg (b, e), 45mg/kg (c, f) and methylcellulose-treated animals (a, d), showing an reduction of APP positive cells for DMF-treated rats. Scale bars indicate 100μm for a-c and 50μm for d-f. (B) Mean numbers of APP positive cells per mm2 sciatic nerve sections as calculated by immunohistochemistry on day 16 p.i. from EAN rats (n = 6/group) receiving orally DMF at different doses (15mg/kg, 45mg/kg/day) and methylcellulose-treated rats. Mean values and SEM are depicted (*p<0,05). The experiment was repeated 2 times with similar results.
